# Safety and early efficacy of involved-field SBRT for nodal oligo-recurrent prostate cancer

**DOI:** 10.3389/fonc.2024.1434504

**Published:** 2024-10-17

**Authors:** Min Ji Koh, Padraig Pilkington, Min Jung Koh, Mary-Kate Lawlor, Michael Creswell, Timothy O’Connor, Alan Zwart, Malika Danner, Deepak Kumar, Simeng Suy, Michael Carrasquilla, Sean P. Collins

**Affiliations:** ^1^ Department of Radiation Medicine, Georgetown University Hospital, Washington, DC, United States; ^2^ Department of Urology, The University of Kansas Health System, Kansas City, KS, United States; ^3^ Biotechnology Research Institute, North Carolina Central University, Durham, NC, United States

**Keywords:** prostate cancer, SBRT, CyberKnife, involved field, common toxicity criteria (CTC), nodal oligo-recurrence

## Abstract

**Purpose:**

Following treatment for localized prostate cancer, a subset of men will develop recurrent disease in the abdominopelvic nodes. For radiation therapy (RT), the optimal treatment volume, fractionation schedule, and dose remain unanswered questions. We report early outcomes for patients treated with involved-field stereotactic body radiation therapy (SBRT) (IF-SBRT) for nodal oligo-recurrent (NOR) prostate cancer.

**Methods:**

Between January 2018 and October 2023, 67 patients with a median age of 75 with NOR prostate cancer treated with 74 courses of IF-SBRT at Georgetown were eligible for this analysis. NOR was defined as any volume of disease that could be safely treated within an IF. All patients were treated with five-fraction IF-SBRT (27.5–35 Gy). The IF treatment volume was defined as the nodal basin containing the gross disease as well as the immediately adjacent basins. Disease progression was defined as a prostate-specific antigen (PSA) rise above the pretreatment baseline or initiation of a second treatment. Local control and progression-free survival were calculated using the Kaplan–Meier method.

**Results:**

Detection of pre-SBRT NOR was ascertained by prostate-specific membrane antigen (PSMA) (38%), fluciclovine (50%), or MRI/CT (12%). Median follow-up was 50 months (1–262). The median pre-salvage PSA was 6.5 ng/mL (range, 0.1–335). The median number of involved nodes was 3 (range, 1–16). The local control at 1 and 2 years was 98% and 93%, respectively. The 1- and 2-year progression-free survival was 78% and 50%, respectively. Twenty percent of treatment courses were followed by acute Grade 2 gastrointestinal (GI) toxicity: diarrhea (9%) and/or nausea (14%). Two patients (3%) experienced late Grade 2 nausea. On univariate analysis, measures of disease volume such as hormone sensitivity (*p* = 0.03), increasing involved node number (*p* = 0.008), and abdominal treatment (*p* = 0.03) were significantly associated with GI toxicity.

**Conclusions:**

With the widespread adoption of PSMA agents, NORs are likely to increase. The optimal combination of local and systemic therapy in this population is unknown. With a favorable toxicity profile, IF-SBRT represents a safe and convenient local therapy treatment option for an elderly patient population. Patient- and treatment-related factors such as a large number of involved nodes and/or abdominal treatment may be associated with an increased risk of GI toxicity.

## Background

The management of nodal oligo-recurrent prostate cancer is an area of active clinical investigation. Prostate cancer has a tropism for lymph nodes, making it a common and, frequently the only, site of metastatic disease (1). The prognosis for men with nodal oligo-recurrent prostate cancer is superior to that for patients with bone metastases (2). In the past, patients were primarily treated with androgen deprivation therapy (ADT) alone (3). Patients receiving ADT with low-volume disease and isolated lymph node metastases fare particularly well with longer remissions (4).

All patients with nodal oligo-recurrences will ultimately progress on ADT despite the initial response. Data are emerging that local radiotherapy may be effective in treating these nodal oligo-recurrences (5). Studies have used several radiotherapy treatment approaches ranging from involved node (focal) stereotactic body radiation therapy (SBRT) to extended nodal conventionally fractionated radiotherapy (ENRT) (1). With the available data, no consensus treatment approach has emerged, with each approach having potential benefits and drawbacks (1).

For patients with disease limited to a few nodal basins, SBRT to the nodal oligo-recurrences may offer long-term local control and increased progression-free survival. SBRT accurately delivers high doses to the target volume while sparing adjacent normal tissues such as the small bowel. SBRT may improve tumor control and reduce treatment-related toxicity through improved target accuracy. This allows for high-dose (>5 Gy per fraction) extremely hypofractionated treatment courses (1–5 fractions) that may be more radiobiologically effective and are certainly more convenient for patients than conventionally fractionated radiotherapy (6).

Most studies until this point have focused on the involved node (focal) SBRT (5). Additional treatment volumes include involved site and involved-field radiotherapy (1). These volumes expand on involved nodes to include an expansion on the gross disease to cover adjacent microscopic diseases. The rationale for these approaches stems from the poor sensitivity of currently available imaging for small-volume nodal disease (<0.5 cm) (7–10), and the adjacent nodal failures are often seen when patients are treated with involved node SBRT (11). Prospective studies with long follow-up including GETUG P07 (12) and PET/CT-Choline-TT (13) have demonstrated very good local and progression-free survival with ENRT and hormonal therapy potentially with increased toxicity (11).

Involved-field radiotherapy involves an expansion to include the involved nodal basin as well as adjacent nodal basins (14). Given the radiobiological advantages of SBRT, it is reasonable to believe that when combined with an involved-field treatment volume with a boost to gross disease and utilization of ADT, the rate of local and distant control could improve (1).

## Methods

### Patient selection

Patients with nodal oligo-recurrent prostate cancer treated with involved-field SBRT at a MedStar Georgetown University Hospital were eligible for inclusion in this analysis. Patients with bone metastases were excluded. Institutional review board (IRB) approval was obtained for this prospective quality-of-life study (IRB 2012-1175).

### SBRT treatment planning and delivery

**Figure 1:**
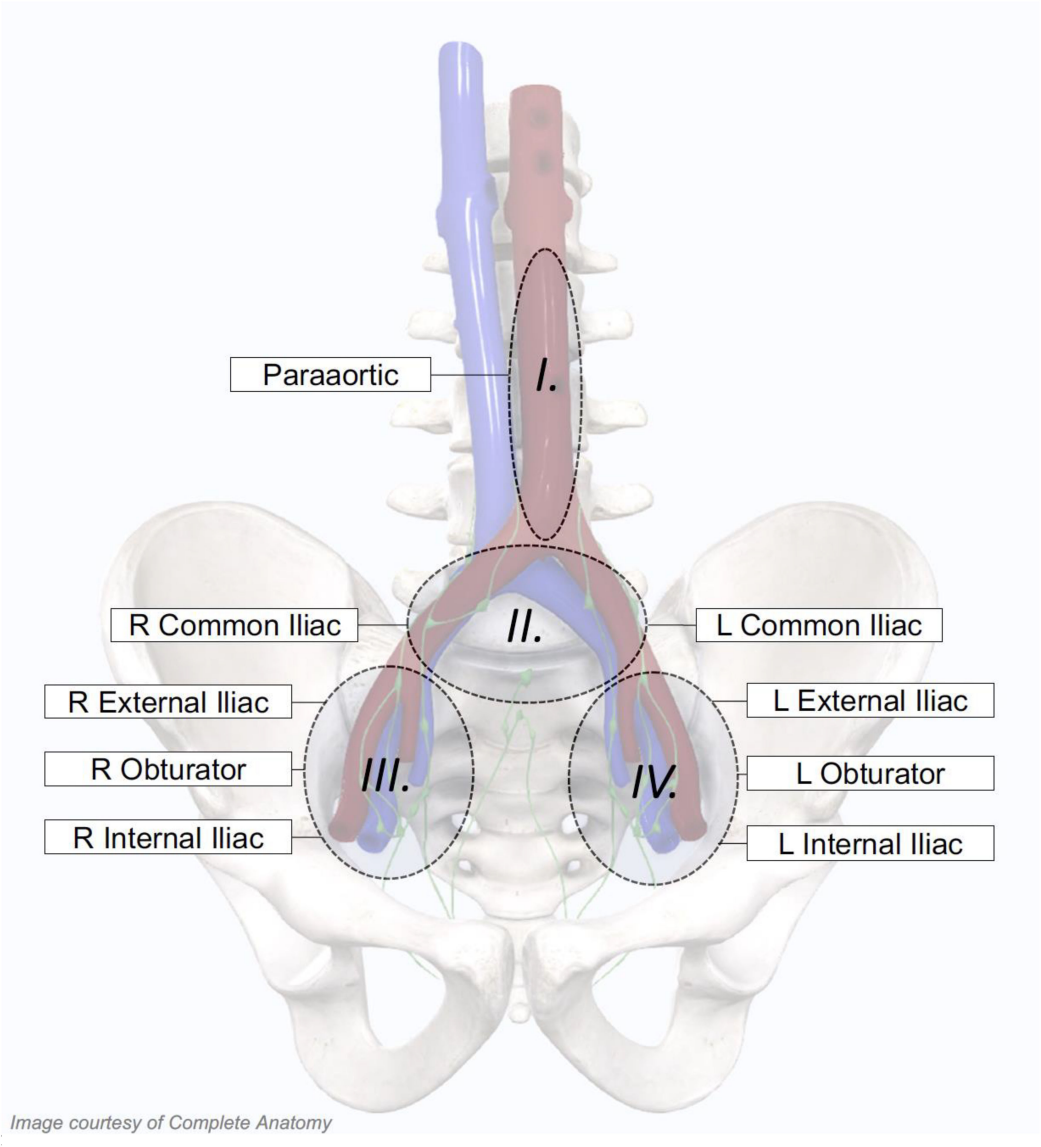
Treatment volumes for individualized involved-field stereotactic body radiation therapy (SBRT). Patient-specific involved fields were utilized, which included specific nodal basins depending on the location of the patients’ PET-positive nodes. For patients with isolated para-aortic involvement, the para-aortic nodal basin alone was treated. If either the left or right common iliac nodal basins were involved, the bilateral common iliac nodal basins were treated. Finally, if the obturator, external iliac, or internal iliac nodal basins were involved on either side, all three unilateral nodal stations were included in the involved field.

Nodal oligo-recurrence was defined as any volume of nodal disease that could be safely treated within an involved field. The planning target volume (PTV) for the involved field was defined as the nodal basin containing the gross nodal disease as well as the immediately adjacent nodal basins (**Figure 1**) (1). Nodal basins were contoured per Radiation Therapy Oncology Group (RTOG) guidelines (15). All patients were treated with five-fraction involved-field SBRT to a dose of 27.5 Gy over 5 to 11 days. Using the linear-quadratic equation with an alpha/beta ratio of 1.5 Gy, the dose was equivalent to 55 Gy in 27–28 fractions. A subset of patients received a prostate-specific membrane antigen (PSMA)**-**guided simultaneous integrated boost (SIB) up to 30–35 Gy. The total dose to the involved nodes ranged from 27.5 to 35 Gy. In general, pelvic-only recurrences were treated with homogeneous volumetric modulated arc therapy (VMAT) with daily cone beam computed tomography (CBCT) guidance (RapidArc, Varian, Palo Alto, CA, USA), and para-aortic (PA)-only recurrences were treated with non-homogeneous robotic SBRT with continuous spine tracking (CyberKnife, Accuray, Sunnyvale, CA, USA) (16). The choice of RT modality, boost dose, and usage of ADT was dictated by the treating radiation oncologist (SPC).

### Follow-up and statistical analysis

Prostate-specific antigen (PSA) and total testosterone levels were obtained before treatment and during routine follow-up visits every 3 months. PSA response was defined as any decrease in the PSA following treatment. Progression was defined as a follow-up PSA that was less than or equal to baseline or re-initiation of treatment. Local recurrence was defined as any new or growing lesion within the treatment field. Oligo-nodal recurrences outside the irradiated field were treated with a second course of involved-field SBRT.

Toxicity was prospectively documented at follow-up visits using the National Cancer Institute (NCI) Common Toxicity Criteria (CTC) version 4.0. Antidiarrheal and antiemetic use were documented at each follow-up. Acute and late toxicity data were defined as any treatment-related toxicity occurring ≤90 days and >90 days following treatment, respectively. The gastrointestinal toxicities analyzed were nausea and diarrhea. In general, Grade 1 toxicity represents minimal side effects not requiring medications for symptom control. Grade 2 toxicity indicates symptoms requiring a medication (i.e., antiemetic or antidiarrheal). Grade 3 indicates complications requiring minor surgical intervention. At each follow-up visit, toxicity events were scored independently for each of the different toxicity types, and the highest gastrointestinal (GI) toxicity was determined for each patient. Actuarial likelihood estimates for GI toxicities were determined using the Kaplan–Meier method. The highest GI toxicity available for each patient was evaluated for the actuarial analysis.

## Results

Patient demographic and clinical characteristics are reported in **Table 1**. Detection of pre-SBRT nodal oligo-recurrence was ascertained by imaging including PSMA (38%), fluciclovine F18 (50%), or MRI/CT (12%) following a PSA rise. The median pre-salvage PSA was 6.5 ng/mL (range, 0.1–335 ng/mL). Of the cases, 86% had hormone-sensitive prostate cancer. The median number of involved nodes was 3 (range, 1–16). In 49% of the cases, involved nodes were boosted to a higher dose. Lower abdominal nodes were included in 58% of treatment fields. Of the treatments, 73% were combined with concurrent hormone therapy. Of the patients, 10% completed a second course of IF-SBRT.

**Table 1:** Demographic and clinical characteristics.

Characteristics	No (%)				*p*-Value^a^
	Patients (n = 67)	Cases (n = 74)	Toxicity (n = 34)	No toxicity (n = 40)	
**Age at treatment** (years), mean ± SD <60 60–69 70–79 >80	75 ± 7.8 2 (3) 15 (22) 30 (45) 20 (30)	75 ± 7.9 2 (3) 16 (22) 33 (45) 23 (31)	74 ± 7.8 2 (6) 6 (18) 17 (50) 9 (26)	76 ± 7.9 0 10 (25) 16 (40) 14 (35)	0.3 0.3
**Race** White Black Other	44 (66) 19 (28) 4 (6)	50 (68) 20 (27) 4 (5)	22 (65) 10 (29) 2 (6)	28 (70) 10 (25) 2 (5)	0.9
**BMI** (kg/m^2^), median (IQR) <18.5 18.5–24.9 25–29.9 30–34.9 35–39.9 40–44.9	28 (26–32) 0 14 (22) 26 (41) 15 (24) 5 (8) 3 (5) (n = 63)	28 (26–33) 0 16 (23) 28 (41) 17 (25) 5 (7) 3 (4) (n = 69)	27 (25–31) 0 8 (26) 11 (35) 8(26) 4 (13) 0 (n = 31)	28 (26–31) 0 8 (21) 17 (45) 9 (24) 1 (3) 3 (8) (n = 38)	0.7 0.8
**Pretreatment PSA**, median (IQR)	6.1 (2.2–18)	6.5 (2.3–18)	8.1 (2.8–29)	5.2 (2.2–14)	0.1
Prior treatment					
Radical prostatectomy Prostate SBRT	20 (30) 63 (94)	20 (27) 70 (95)	10 (14) 32 (43)	10 (14) 38 (51)	0.8 >0.99
**Current ADT** Yes No	50 (75) 17 (15)	54 (73) 20 (27)	28 (82) 6 (18)	26 (65) 14 (35)	0.1
**Pre-SBRT imaging** PSMA Axumin MRI/CT	25 (37) 33 (49) 9 (13)	28 (38) 37 (50) 9 (12)	14 (41) 16 (47) 4 (12)	14 (35) 21 (52) 5 (12)	0.9
**Hormone sensitivity** Castrate sensitive Castrate resistant	57 (85) 10 (15)	64 (86) 10 (14)	26 (76) 8 (24)	38 (95) 2 (5)	**0.04**
**Target volumes** Boost No boost	33 (49) 34 (51)	36 (49) 38 (51)	16 (47) 18 (53)	20 (50) 20 (50)	0.8
**# of involved lymph nodes** Mean ± SD Median (IQR)	4.2 ± 3.7 3 (2-6) (n = 66)	4.2 ± 3.6 3 (2-6) (n = 73)	5.6 ± 4.1 4 (2-8) (n = 33)	3.2 ± 2.8 2 (1-4)	**0.004**
**Location of radiation** Abdomen Pelvis Abdomen + pelvis	17 (25) 28 (42) 22 (33)	20 (27) 31 (42) 23 (31)	12 (35) 9 (26) 13 (38)	8 (20) 22 (55) 10 (25)	**0.045**

BMI, body mass index; IQR, interquartile range; PSA, prostate-specific antigen; SBRT, stereotactic body radiotherapy; ADT, androgen deprivation therapy; PSMA, prostate-specific membrane antigen.

a p-Values for the comparison between patients with and without toxicity were calculated using Wilcoxon rank sum test and Fisher’s exact test for non-normally distributed continuous and categorical variables, respectively. The bolded values indicate that they are statistically significant.

Cancer control outcomes are shown in **Table 2**. The median follow-up for all patients was 50 months (1–262 months). Of the cases, 80% had a PSA response following treatment. The local control at 1 and 2 years was 98% and 93%, respectively. The 1- and 2-year progression-free survival was 78% and 50%, retrospectively. In the 20 patients who did not receive ADT, the progression-free survival at 1 and 2 years was 56% and 31%, respectively. Of the 54 patients who were treated in the pelvis, there was one local recurrence and 14 distance recurrences. Two of the distant recurrences were in the contralateral pelvis. Of the 43 patients who were treated in the abdomen, there were no local failures and 11 distant recurrences, including one in the upper abdomen. The overall survival at 1 and 2 years was 99% and 96%, respectively. Three patients died from prostate cancer.

**Table 2:** Cancer control outcomes.

Characteristics	No (%)				*p*-Value^a^
	Patients (n = 67)	Cases (n = 74)	Toxicity (n = 34)	No toxicity (n = 40)	
**PSA response** Yes No	50/64 (78) 14/64 (22)	56/70 (80) 14/70 (20)	25/32 (78) 7/32 (22)	31/38 (82) 7/38 (18)	0.8
**Local control** 1 year 2 years	41/42 (98) 24/26 (92)	47/48 (98) 26/28 (93)	17/17 (100) 10/10 (100)	30/31 (97) 16/18 (89)	>0.99 0.5
**Progression-free survival** 1 year 2 years	45/59 (76) 19/39 (49)	51/65 (78) 22/44 (50)	24/29 (83) 10/20 (50)	27/36 (75) 12/24 (50)	0.6 >0.99
**Overall survival** 1 year 2 years	66 (99) 64 (96)	73 (99) 71 (96)	34 (100) 33 (97)	39 (98) 38 (95)	>0.99 >0.99

PSA, prostate-specific antigen.

a p-Values for the comparison between patients with and without toxicity were calculated using Wilcoxon rank sum test and Fisher’s exact test for non-normally distributed continuous and categorical variables, respectively.

Common Terminology Criteria for Adverse Events (CTCAE)-graded GI toxicities are shown in **Table 3**. Of the treatment courses, 20% were followed by acute Grade 2 GI toxicity: diarrhea (9%) and/or nausea (14%). Two patients (3%) experienced late Grade 2 nausea. No Grade 3+ side effects occurred at any time following treatment. On univariate analysis (**Table 4**), measures of disease volume such as hormone sensitivity, increasing involved node number, and abdominal treatment were significantly associated with GI toxicity. No demographic or clinical characteristics were significant in the multivariate analysis (**Table 5**).

**Table 3:** Toxicity/adverse effects.

Adverse effects	No (%)		
	Cases (n = 74)		
	Diarrhea	Nausea	Combined
Toxicity None Grade 1 Grade 2 Grade 3	53 (72) 14 (19) 7 (9) 0	55 (74) 9 (12) 10 (14) 0	40 (54) 19 (26) 15 (20) 0
Acute Grade 1 Grade 2 Late Grade 1 Grade 2	14 (19) 7 (9) 0 0	9 (12) 8 (11) 0 2 (3)	19 (26) 13 (18) 0 2 (3)

**Table 4:** Univariate logistic regression model predicting the risk of oligometastatic prostate cancer with toxicity vs. no toxicity.

	β ± s.e.	Odds ratio	*p*-Value
Age (years) Age (Ref: <60) 60–69 70–79 >80	−0.032 ± 0.03 −17.1 ± 1,697 −16.5 ± 1,697 −17.0 ± 1,697	0.97 3.83e−8 6.79e−8 4.12e−8	0.3 >0.99 >0.99 >0.99
Race (Ref: White) Black Other	0.24 ± 0.53 0.24 ± 1.03	1.27 1.27	0.7 0.8
BMI BMI (Ref: 18.5–24.9) 25.0–29.9 30.0–34.9 35.0–39.9 ≥40.0	−0.034 ± 0.04 −0.43 ± 0.63 −0.12 ± 0.70 1.39 ± 1.23 −1.66 ± 1,385	0.97 0.65 0.89 4.00 6.39e−8	0.4 0.5 0.9 0.3 0.99
Pretreatment PSA	0.016 ± 0.01	1.02	0.1
PSA response (Ref: Yes) No	−0.22 ± 0.60	0.8	0.7
Current ADT (Ref: No) Yes	0.92 ± 0.56	2.51	0.1
Prior treatment Radical prostatectomy (Ref: No) Yes Prostate SBRT (Ref: No) Yes	0.22 ± 0.52 −0.17 ± 1.03	1.25 0.84	0.7 0.9
Pre-SBRT imaging (Ref: PSMA) Axumin MRI/CT	−0.27 ± 0.50 −0.22 ± 0.77	0.76 0.80	0.6 0.8
Hormone sensitivity (Ref: Castrate sensitive) **Castrate resistant**	1.77 ± 0.83	5.85	**0.03**
Target volume (Ref: No boost) Boost	−0.12 ± 0.47	0.89	0.8
**# of lymph nodes**	0.20 ± 0.08	1.22	**0.008**
Location of radiation (Ref: Abdomen) Abdomen + pelvis **Pelvis**	−0.14 ± 0.62 −1.3 ± 0.60	0.87 0.27	0.8 **0.03**

BMI, body mass index; PSA, prostate-specific antigen; ADT, androgen deprivation therapy; SBRT, stereotactic body radiotherapy. The bolded values indicate that they are statistically significant.

**Table 5:** Multivariate logistic regression model predicting the risk of oligometastatic prostate cancer with toxicity vs. no toxicity.

	Odds ratio (*p*-value)			
	Univariate	Multivariate		
# of lymph nodes	1.22 **(0.008)**	3.6 (0.2)	2.3 (0.4)	0.7 (0.7)
Current ADT: inhibitor (Ref: No)	6.84 (0.02)	6.3 (0.03)		
Castrate resistant (Ref: Castrate sensitive)	5.85 (0.03)		5.1 (0.06)	
Location: pelvis (Ref: Abdomen)	0.27 (0.03)			0.4 (0.2)

ADT, androgen deprivation therapy.

## Discussion

Our institutional experience adds to the growing body of evidence supporting the safety and effectiveness of SBRT for nodal oligo-recurrent prostate cancer. Our early PSA outcomes have been favorable with low rates of early local recurrence considering the inclusion of patients with castrate-resistant disease and relatively low biologically effective dose (BED) (17). As with other SBRT series and other radiation therapy modalities, recurrences were common in higher nodal stations (18). Considering that our series included a low rate of pretreatment PSMA imaging and a relatively high baseline PSA/nodal burden, our suboptimal 50% actuarial 2-year biochemical failure-free survival rate is not unexpected. It is hoped that with salvage at lower baseline PSAs with pretreatment PSMA staging, this percentage would increase.

Toxicity following lower abdominal/upper pelvic SBRT was similar to that following conventionally fractionated radiation therapy at these sites (11). Acute and late Grade 2 GI toxicity were observed in 20% and 3%, respectively. Antiemetic or antidiarrheal utilization was the most common Grade 2 toxicity. There were no Grade 3 toxicities. Knowledge of these GI toxicities and their resolution with conservative management will enable clinicians to relieve patient concerns. Our outcomes are similar to those of para-aortic nodes treated with conventionally fractionated radiation therapy and pelvic nodes treated with SBRT (11, 19–22).

We argue against proposing a strict numerical cut-off for the number of nodes to qualify for nodal oligo-recurrent nodal disease. Increased nodal involvement was associated with increased GI toxicity. However, GI toxicity was low-grade and short-lived in most cases. Instead, we advocate for any volume of nodal disease that can be safely treated with curative intent. This approach preserves the rationale for treating nodal recurrent disease, which is to halt or delay widespread disease progression while maximizing the number of patients who may benefit. Because the rationale for treatment of nodal disease is diminished if cancer has already spread past the lymph nodes, one must be relatively certain that no other systemic disease is present. For this reason, we recommend oligo-recurrence only be defined with the use of advanced PET imaging, preferably PSMA PET, if available, which has been shown to outperform other agents in this clinical setting (23).

Our study has several limitations. First, it is limited by the retrospective nature of the analysis. However, subjects were accrued consecutively to a prospective quality of life (QOL) study, and all data were collected prospectively in a centralized database, therefore limiting selection and reporting bias present in pure retrospective studies. Second, the study was initiated prior to the widespread availability of PSMA PET scans in the United States. Therefore, not all patients had a pretreatment PSMA scan, so the extent of the disease was likely underestimated. If all patients had received a pretreatment PSMA PET, it is likely that cancer control would have been improved (24).

## Conclusions

Involved-field SBRT for nodal oligo-recurrent prostate cancer is well-tolerated. Local recurrence in the radiation field was uncommon. As shown previously, out-of-field recurrences were common in superior nodal stations but potentially treatable (25, 26). Rates of late GI toxicity are comparable to those of alternative radiation therapy. Patient- and treatment-related factors such as a large number of involved nodes and/or abdominal treatment may be associated with an increased risk of GI toxicity.

## Data Availability

The raw data supporting the conclusions of this article will be made available by the authors, without undue reservation.
